# Clinical features and short-term outcomes in children with *Mycoplasma pneumoniae* encephalitis

**DOI:** 10.1186/s12879-026-13253-2

**Published:** 2026-04-04

**Authors:** Yong-zhan Zhang, Ming-xin Xing, Ling-yun Guo, Hai-juan Xiao, Zhen-zhen Dou, Liang Zhu, Xin Guo, Tian-ming Chen, Bing Hu, Hui-li Hu, Xiu-wei Zhuo, Zheng Li, Quan Wang, Hui Xiong, Gang Liu

**Affiliations:** 1 Department of Infectious Diseases, Beijing Children’s Hospital, Capital Medical University, National Center for Children’s Health, National Center for Children’s Infectious and Allergic Diseases Surveillance, Beijing Research Center for Respiratory Infectious Diseases, Beijing Key Laboratory of Core Technologies for the Prevention and Treatment of Emerging Infectious Diseases in Children, Key Laboratory of Major Diseases in Children, Ministry of Education, Beijing, 100045 China; 2 Department of Neurology, National Center for Children’s Health, Beijing Children’s Hospital, Capital Medical University, Beijing, 100045 China; 3 Department of Paediatric Intensive Care Unit, National Center for Children’s Health, Beijing Children’s Hospital, Capital Medical University, Beijing, 100045 China

**Keywords:** *Mycoplasma pneumoniae* encephalitis, Clinical features, Prognostic factors, Paediatric

## Abstract

**Background:**

In recent years, the infection rate of *Mycoplasma pneumoniae* in children has gradually increased. *Mycoplasma pneumoniae* encephalitis (MPE) seriously threatens children’s health, but studies on its clinical characteristics and prognosis are limited. This study summarized the clinical features of paediatric MPE and explored its prognostic factors.

**Methods:**

A retrospective single-center study was conducted on children with MPE who were treated between January 2016 and June 2024. All children underwent Glasgow Outcome Scale (GOS) assessment at 6 months after diagnosis. Patients with GOS scores of 4–5 were assigned to the good outcome group, and those with scores of 1–3 to the poor outcome group. Early onset was defined as the onset of neurological symptoms within 7 days of fever onset, and late onset as symptoms after 7 days. Characteristics and prognostic factors were subsequently analysed.

**Results:**

Among 71 children, the median age was 7 years; 43 males and 28 females were affected, and 36 had winter onset. Thirty-one patients had early-onset disease, and 40 patients had late-onset disease. Compared with early-onset patients, late-onset patients had a younger median age, a greater proportion of patients with cough symptoms, a lower proportion of patients with vomiting symptoms, and a greater proportion of patients with elevated D-dimer levels, elevated serum IgM levels and severe pneumonia. Nine children had poor prognoses, with a GOS score of 3. Multivariate analysis revealed that a reduced level of consciousness at onset (odds ratio [OR] = 20.978; *P* = 0.006), elevated serum IgG levels (OR = 23.813; *P* = 0.030), and invasive mechanical ventilation (OR = 39.275; *P* = 0.005) were independent risk factors affecting the prognosis of children with MPE. The timing of anti-*Mycoplasma pneumoniae* or immunomodulatory treatment was not significantly correlated with prognosis.

**Conclusions:**

Most children with MPE have a good prognosis. A reduced level of consciousness at onset, elevated serum IgG levels, and invasive mechanical ventilation may indicate a poor prognosis and help identify severe cases early. Future research should focus on the pathogenesis of MPE and explore novel therapeutic approaches to improve the prognosis of children with MPE.

**Supplementary Information:**

The online version contains supplementary material available at 10.1186/s12879-026-13253-2.

## Introduction

*Mycoplasma pneumoniae* has become one of the most common pathogens causing respiratory tract infections in children. It can also cause extrapulmonary injuries. Among the nervous system diseases caused by *Mycoplasma pneumoniae*, *Mycoplasma pneumoniae* encephalitis (MPE) is the most common type, accounting for 5% to 10% of childhood encephalitis cases [1, 2]. With the increase in the infection rate of *Mycoplasma pneumoniae* in recent years, the incidence of MPE has also been increasing annually. Owing to the atypical symptoms of *Mycoplasma pneumoniae* infection and the lack of obvious clinical and imaging features, early infections are easily underestimated. The pathogenic mechanism of *Mycoplasma pneumoniae* is complex and may include direct damage mediated by invasion and inflammatory factors, indirect damage caused by the host immune response, and vascular occlusion. The specific mechanism is still not fully understood [3–5]. In addition, although some children recover completely, 20% to 64% of affected children develop neurological dysfunctions, status epilepticus, and severe neurological sequelae, which pose a serious threat to their health [6]. Therefore, identifying MPE as early as possible, understanding the factors affecting patient prognosis, and providing active treatment are crucial. However, few studies have comprehensively analysed the clinical characteristics and prognostic factors of MPE in children. This study summarizes the clinical characteristics and auxiliary examination data of children with MPE, explores the prognostic factors of MPE in children, and hopes to provide valuable insights for clinicians in the diagnosis, treatment, and prognosis prediction of this challenging disease.

## Patients and methods

### Study design

**Fig. 1:**
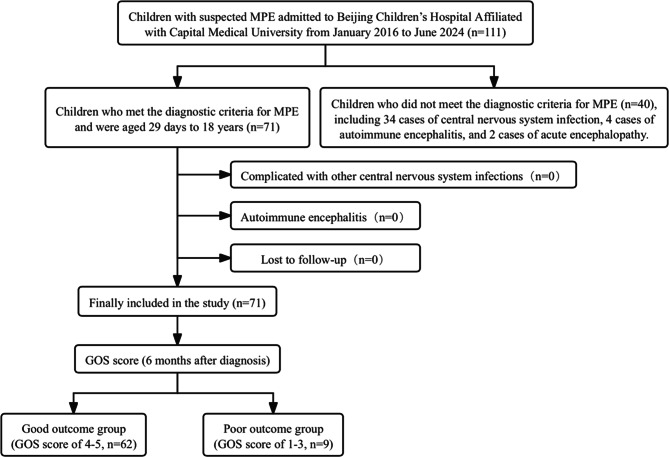
Flowchart of patient selection. *GOS* Glasgow Outcome Scale, *MPE Mycoplasma pneumoniae* encephalitis

This study was a retrospective clinical study that consecutively enrolled patients with MPE who were treated at Beijing Children’s Hospital affiliated with Capital Medical University from January 2016 to June 2024. The inclusion criteria were as follows: met the diagnostic criteria for MPE and were aged 29 days to 18 years. The exclusion criteria included complications of other central nervous system infections, autoimmune encephalitis, and lost to follow-up. The detailed process of patient selection is shown in Fig. 1. All patients underwent Glasgow Outcome Scale (GOS) assessment at 6 months after diagnosis. Patients with a GOS score of 4 or 5 were categorized into the good outcome group, whereas patients with a score ranging from 1 to 3 were classified into the poor outcome group [7]. All clinical data, including imaging examinations and cerebrospinal fluid (CSF) analyses, were obtained prior to the initiation of steroid or intravenous immunoglobulin (IVIG) treatment. The criteria for severe *Mycoplasma pneumoniae* pneumonia (MPP) referred to the *Evidence-based Guidelines for the Diagnosis and Treatment of Mycoplasma pneumoniae Pneumonia in Children (2023)* [8]. 

### Diagnostic criteria for MPE

The diagnosis of MPE is based mainly on the diagnostic criteria proposed by Bitnun [9]. The diagnosis is divided into two levels, namely, the diagnosis of encephalitis and the judgment of the degree of correlation between *Mycoplasma pneumoniae* and encephalitis. Encephalitis is defined as encephalopathy (disturbance of consciousness lasting ≥ 24 h, including drowsiness, extreme irritability, or significant changes in personality or behaviour) with two or more of the following manifestations: fever (body temperature ≥ 38 °C), seizures, focal nerve abnormalities, increased cerebrospinal fluid cell count (white blood cells > 5 × 10^9^/L), and changes in electroencephalogram (EEG) or abnormal imaging findings consistent with encephalitis. MPE can be clinically diagnosed after the diagnostic conditions for encephalitis, such as positive CSF culture and/or polymerase chain reaction (PCR), with or without a positive serological test, or only a positive throat swab culture and/or PCR with a positive serological test, are met. Early onset is defined as the onset of neurological symptoms within 7 days of fever, and late onset is defined as the onset of neurological symptoms after 7 days of fever [10, 11]. 

### Statistical analysis

Normally distributed data are described as the means ± standard deviations. Nonnormally distributed data are described as medians (ranges), and categorical variables are described as counts (frequencies). Quantitative indicators are compared via analysis of the Mann‒Whitney U test according to the data distribution; classification indicators are compared via the chi‒square test. In accordance with the nature of the variables, the Pearson chi-square test, continuous correction or Fisher’s exact test was used for single-factor analysis. Prognostic factors with *P* < 0.05 in univariate analysis were adjusted in multivariate analysis, and binary logistic regression was used for multivariate analysis. *P* < 0.05 was considered statistically significant. All the statistical analyses were conducted primarily via SPSS 26.0 (SPSS Inc., Chicago, IL, USA).

## Results

### Clinical characteristics

**Fig. 2:**
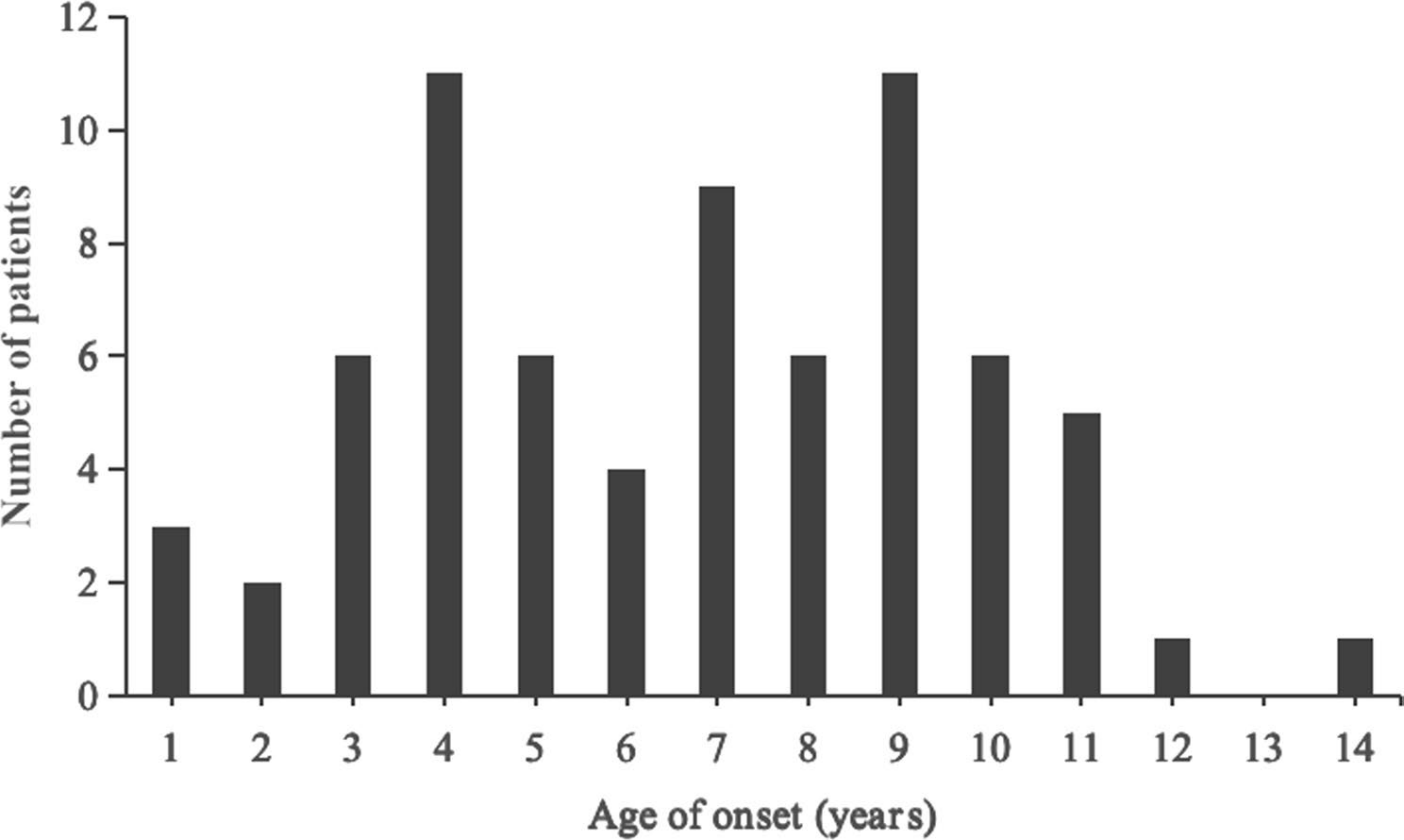
Age distribution at onset in all patients with *Mycoplasma pneumoniae* encephalitis

**Fig. 3:**
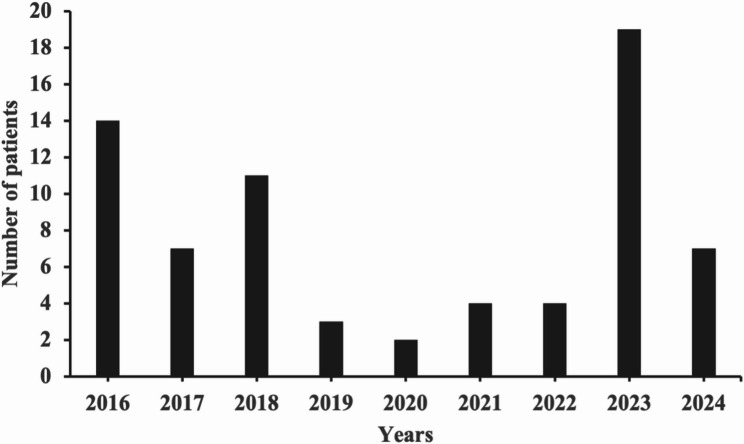
Distribution of onset years among all patients with *Mycoplasma pneumoniae* encephalitis

**Fig. 4:**
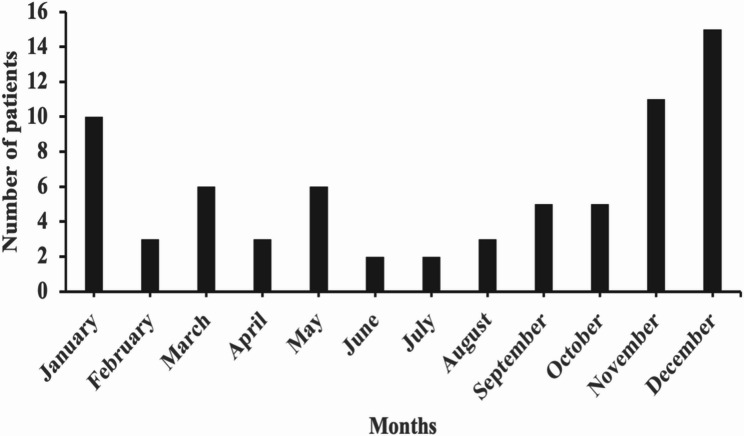
Distribution of onset months among all children with *Mycoplasma pneumoniae* encephalitis

A total of 71 children with MPE were enrolled in the study, with a median age of 7 years (1–14 years) (Fig. 2). There were 43 males and 28 females, with a male-to-female ratio of approximately 1.5:1. The number of MPE cases was small during the COVID-19 pandemic, and a peak incidence occurred in 2023 after the pandemic (Fig. 3). Winter onset was observed in 36 patients (50.7%) (Fig. 4). The average length of hospital stay was 19 days. The most common neurological manifestations were headache (56.3%), vomiting (42.3%), and seizures (35.2%), whereas the most common extraneurological manifestations were fever (95.8%) and cough (76.1%). A reduced level of consciousness at disease onset occurred in 9 patients (12.7%), 7 patients (9.9%) received invasive mechanical ventilation, and status epilepticus developed in 6 patients (8.5%). Other neurological manifestations included stroke in 4 patients, neurogenic bladder in 2 patients, neurogenic shock in 2 patients, hearing impairment in 1 patient, and diaphragmatic paralysis in 1 patient. The extraneurological manifestations included liver dysfunction in 31 patients, myocardial injury in 18 patients, coagulation dysfunction in 9 patients, anaemia in 8 patients, rash in 4 patients, hypokalaemia in 4 patients, hyponatremia in 3 patients, oral mucositis in 1 patient, and thrombophlebitis in 1 patient.

All patients were positive for *Mycoplasma pneumoniae* nucleic acid in their throat swabs and for *Mycoplasma pneumoniae*-IgM in their serum. Cerebrospinal fluid was positive for *Mycoplasma pneumoniae*-IgM in 7 patients (9.9%), and cerebrospinal fluid was positive for *Mycoplasma pneumoniae*-nucleic acid in 4 patients (5.6%). The median initial white blood cell (WBC) count of all patients was 10.5 (2.0–42.5) ×10^9^/L, and the median lactate dehydrogenase (LDH) value was 277 (137–2558) U/L. There were 34 patients (47.9%) with elevated C-reactive protein (CRP), 35 patients (49.3%) with abnormal liver function, 18 patients (25.4%) with abnormal renal function, and 40 patients (56.3%) with elevated D-dimer. Fifty-four patients (76.1%) had CSF WBC counts > 5 × 10^6^/L, and 31 patients (43.7%) had elevated CSF protein levels. Forty-eight patients (67.6%) had concurrent pneumonia, among whom 18 patients (25.4%) had severe pneumonia, 7 patients (9.9%) had concurrent bronchitis, and 16 patients (22.5%) had no abnormalities on chest computed tomography (CT).

Thirty-one patients (43.7%) had early-onset disease, and 40 patients (56.3%) had late-onset disease. The clinical characteristics of all patients, as well as those with early-onset and late-onset disease, are summarized in Table 1. Compared with early-onset patients, late-onset patients had a younger median age (5 years vs. 8 years, *P* < 0.001), a greater proportion of patients with cough symptoms (87.5% vs. 61.3%, *P* = 0.010), a lower proportion of patients with vomiting symptoms (30.0% vs. 58.1%, *P* = 0.018), and a greater proportion of patients with elevated D-dimer (67.5% vs. 41.9%, *P* = 0.031), elevated serum IgM (42.5% vs. 9.7%, *P* = 0.002) and severe pneumonia (35.0% vs. 12.9%, *P* = 0.034).

**Table 1:** Clinical characteristics of all MPE patients

Parameters	All patients (*n* = 71)	Onset type		
		Early-onset (*n* = 31)	Late-onset (*n* = 40)	*P* value
Age (years)	7 (1–14)	8 (3–14)	5 (1–11)	<0.001
Male	43 (60.6)	21 (67.7)	22 (55.0)	0.276
Fever	69 (97.2)	29 (93.5)	40 (100)	0.187
Cough	54 (76.1)	19 (61.3)	35 (87.5)	0.010
Headache	40 (56.3)	21 (67.7)	19 (47.5)	0.088
Vomiting	30 (42.3)	18 (58.1)	12 (30.0)	0.018
Seizure	25 (35.2)	13 (41.9)	12 (30.0)	0.296
A reduced level of consciousness at onset	9 (12.7)	3 (9.7)	6 (15.0)	0.757
Initial WBC count (×10^9^/L)	10.5 (2.0-42.5)	10.1 (2.0-42.5)	10.7 (4.0-27.5)	0.444
Neutrophil percentage (%)	68.3 (32.8–96.5)	67.3 (32.8–96.5)	69.9 (41.3–95.0)	0.340
Lymphocyte percentage (%)	20.7 (1.7–53.1)	26.4 (1.7–53.1)	18.9 (2.0-46.3)	0.083
CRP elevation	34 (47.9)	11 (35.5)	23 (57.5)	0.066
LDH (U/L)	277.0 (137.0-2558.0)	244.0 (145.0-1099.0)	289.0 (137.0-2558.0)	0.057
Abnormal liver function	35 (49.3)	12 (38.7)	23 (57.5)	0.116
Abnormal renal function	18 (25.4)	7 (22.6)	11 (27.5)	0.637
D-dimer elevation	40 (56.3)	13 (41.9)	27 (67.5)	0.031
Serum IgA elevation	11 (15.5)	3 (9.7)	8 (20.0)	0.401
Serum IgG elevation	27 (38.0)	10 (32.3)	17 (42.5)	0.347
Serum IgM elevation	20 (28.2)	3 (9.7)	17 (42.5)	0.002
Serum IgE elevation	35 (49.3)	14 (45.2)	21 (52.5)	0.419
Initial WBC count of CSF (×10^6^/L)	28 (0-1323)	56 (0-483)	20 (0-1323)	0.461
Glucose of CSF (mmol/L)	3.7 (1.99–9.01)	3.8 (2.0-5.8)	3.7 (2.3-9.0)	0.415
Protein of CSF (mg/L)	400.5 (28.0-3041.0)	480.0 (111.0-1045.0)	356.5 (28.0-3041.0)	0.485
Chloride of CSF (mmol/L)	124.2 (102.5–133.0)	123.8 (109.0-131.0)	125.0 (103.0-133.0)	0.651
Severe pneumonia	18 (25.4)	4 (12.9)	14 (35.0)	0.034
Abnormal EEG	23 (32.4)	12 (38.7)	11 (27.5)	0.491
Abnormal signal on cranial MRI	51 (71.8)	22 (71.0)	29 (72.5)	0.887

*CRP* C-reactive protein, *CSF* cerebrospinal fluid, *EEG* electroencephalogram, *Ig* immunoglobulin, *LDH* lactate dehydrogenase, *MPE Mycoplasma pneumoniae* encephalitis, *MRI* magnetic resonance imaging, *WBC* white blood cell

### Imaging findings

**Fig. 5:**
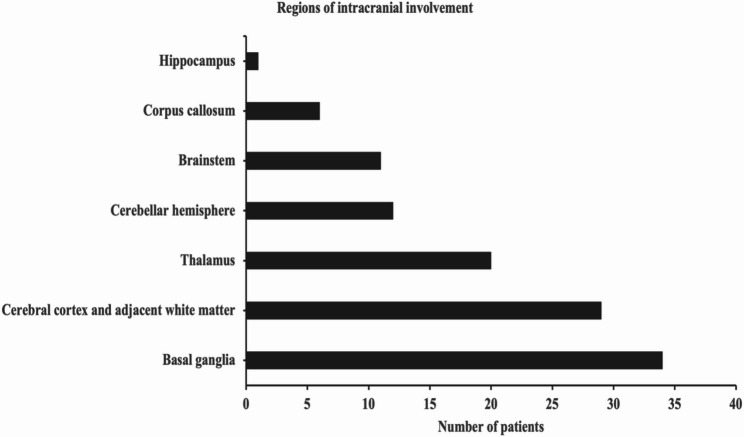
Regions involved on cranial MRI in all patients with *Mycoplasma pneumoniae* encephalitis. *MRI* magnetic resonance imaging

**Fig. 6:**
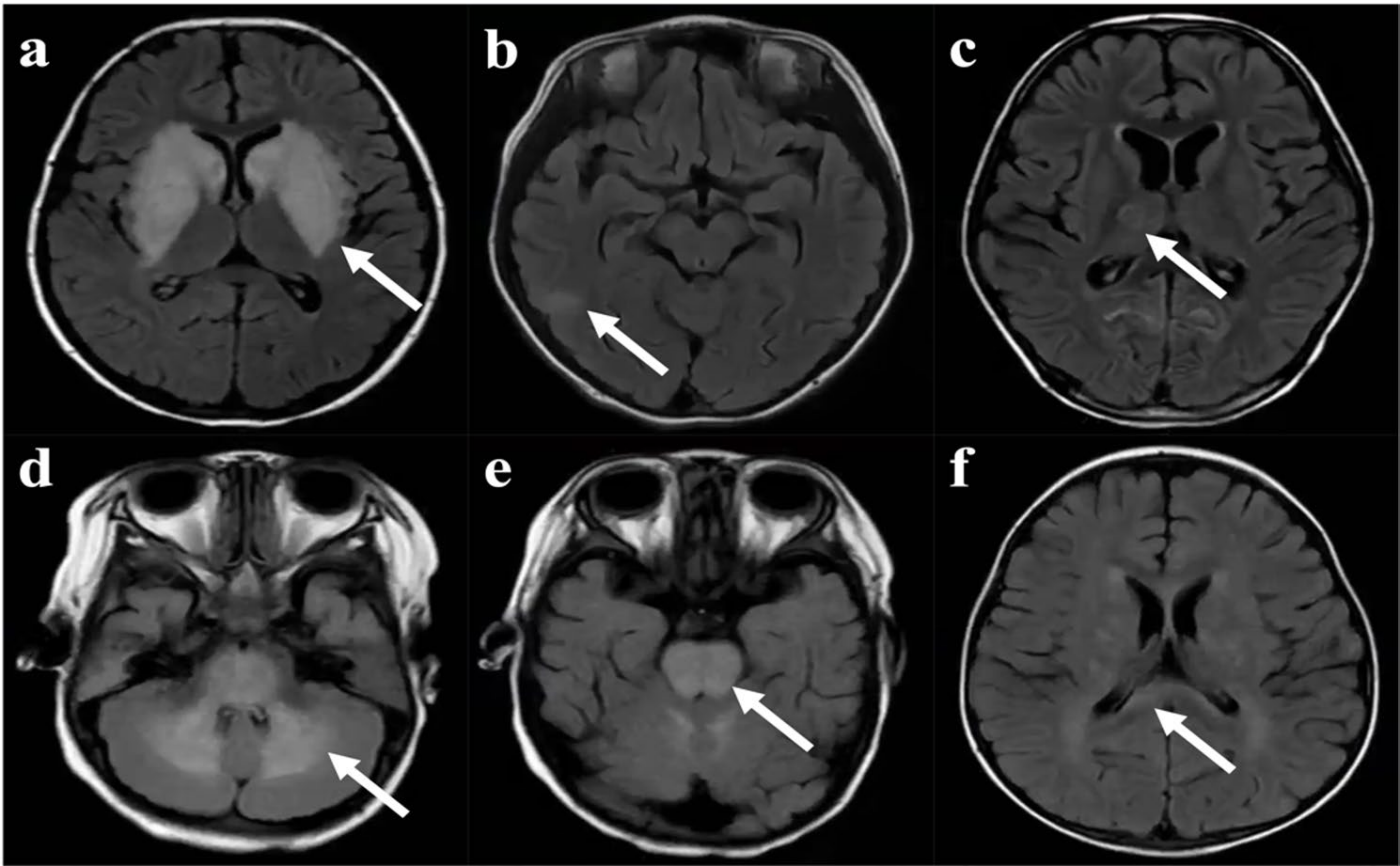
Brain MRI of patients with *Mycoplasma pneumoniae* encephalitis showing lesions in different locations. (**a**) Basal ganglia, T2 FLAIR, axial scan; (**b**) Cerebral cortex and adjacent white matter, T2 FLAIR, axial scan; (**c**) Thalamus, T2 FLAIR, axial scan; (**d**) Cerebellar hemisphere, T2 FLAIR, axial scan; (**e**) Brainstem, T2 FLAIR, axial scan; (**f**) Corpus callosum, T2 FLAIR, axial scan. *FLAIR* Fluid-Attenuated Inversion Recovery, *MRI* magnetic resonance imaging

In this study, all patients underwent cranial magnetic resonance imaging (MRI). There were 51 patients (71.8%) whose cranial MRI results were abnormal, and the intracranial lesions involved mainly the basal ganglia, thalamus, cerebral cortex and adjacent white matter, brainstem, cerebellar hemisphere, corpus callosum, hippocampus, etc (Figs. 5 and 6). The most common site of intracranial involvement was the basal ganglia (47.9%). Cranial MRI of 35 patients (49.3%) revealed that at least 2 sites were involved.

### Treatment

Sixty-nine patients (97.2%) were treated with azithromycin, and the median time from onset to regular use of azithromycin was 6 (0–30) days. The number of patients treated with tetracycline or quinolone antibiotics was 12 (16.9%) and 11 (15.5%), respectively. Sixty-one patients (85.9%) were treated with methylprednisolone, and the median time from onset to first use of methylprednisolone was 10 (2–29) days. Among them, 23 patients (32.4%) received high-dose methylprednisolone shock therapy. Fifty-two patients (73.2%) were treated with human immunoglobulin, and the median time from onset to first treatment was 12 (3–27) days.

Nine children (12.7%) had poor prognoses, all with a GOS score of 3. Among them, 6 had somatic motor dysfunction and language impairment, 2 had only somatic motor dysfunction, and 1 had postencephalitic epilepsy treated with neurostimulation device implantation. There were no deaths or cases of vegetative state. Sixty-two children (87.3%) had good prognoses, including 11 (15.5%) with a GOS score of 4 and 51 (71.8%) with a GOS score of 5. The univariate analysis of prognostic factors is shown in Supplementary Material Table 1. Invasive mechanical ventilation, a reduced level of consciousness at onset, and elevated serum IgG levels are risk factors associated with prognosis. Multivariate analysis revealed that invasive mechanical ventilation, a reduced level of consciousness at onset, and elevated serum IgG levels were independent risk factors affecting the prognosis of children with MPE (Table 2). There was no significant correlation between the time from onset to first anti-*Mycoplasma pneumoniae* treatment or immunomodulatory treatment and patient prognosis.

**Table 2:** Multivariate analysis of factors associated with prognosis in all MPE patients (*n* = 71)

Factors	Odds Ratio (95% CI)	*P* value
A reduced level of consciousness at onset	20.978 (2.345–187.627)	0.006
Elevated serum IgG levels	23.813 (1.369–414.125)	0.030
Invasive mechanical ventilation	39.275 (3.041–507.274)	0.005

A total of 3 predictors were included in the model, with 9 cases of poor outcomes among 71 patients. The number of events per variable (EPV) was calculated as 9/3 = 3 (recommended threshold: EPV ≥ 10). Multicollinearity test showed that the variance inflation factor (VIF) values of all variables were < 5, indicating no significant multicollinearity. Model goodness-of-fit assessment results: Nagelkerke R² = 0.547, Hosmer-Lemeshow test χ² = 4.348, *P* = 0.114 (*P* > 0.05), suggesting a good model fit. *CI* confidence interval, *Ig* immunoglobulin

## Discussion

MPE is the most common type of nervous system disease caused by *Mycoplasma pneumoniae* and poses a serious threat to the life and health of children [1, 2]. This single-center retrospective study investigated the clinical characteristics, laboratory results, treatment regimens, and prognostic factors of childhood MPE in a Chinese cohort. Understanding these aspects is crucial for disease recognition, early identification of severe cases, and optimization of management strategies.

In this study, the median age of the 71 children was 7 years, and 50.7% of the cases occurred in winter, which is similar to other paediatric cohorts [1, 12, 13]. Notably, there were no infant cases, and children aged 3–11 years accounted for 83.1% of all cases, indicating that preschool-aged and school-aged children are more susceptible. This may be related to increased social interaction leading to greater exposure and more mature immune function in this age group. MPE has diverse clinical manifestations, with most children presenting with meningeal irritation symptoms and signs such as headache, vomiting, and neck stiffness. Some children may experience headache and vomiting as the initial symptoms, and respiratory infection symptoms may also appear during the course of the disease, which are similar to those of viral encephalitis or autoimmune encephalitis [12, 13]. The most common neurological manifestations in this study were headache, vomiting, and seizures, whereas the most common extraneurological manifestations were fever and cough. However, not all children have central nervous system or pulmonary symptoms and signs, which poses challenges for early diagnosis.

A study involving 330 children with encephalitis reported that 16.4% developed epilepsy. Significant risk factors for postencephalitic epilepsy include recurrent seizures, status epilepticus, severe disturbance of consciousness, focal neurological signs, and neurological deterioration during hospitalization. Patients with abnormal EEG, severe cerebral dysfunction, and focal cortical abnormalities on neuroimaging also had higher epilepsy incidence rates [14]. Lin et al. reported that nearly 50% of MPE children experienced seizures, approximately 40% of which were intractable, with acute-phase seizures being a critical factor associated with prognosis [6]. Early studies reported high mortality in children with status epilepticus associated with MPE [15], whereas a recent Chinese study suggested that early diagnosis and treatment of MPE with status epilepticus could lead to relatively good outcomes [16]. In this study, 35.2% of patients had seizures, including 24% of patients with status epilepticus. After aggressive treatment, only 1 patient had poor outcomes, manifested as the inability to move or communicate independently, with no seizures after discharge. Additionally, 12.7% of the children developed a reduced level of consciousness at onset, and 9.9% required invasive mechanical ventilation. Multivariate analysis revealed that a reduced level of consciousness at onset and the need for invasive mechanical ventilation were independent risk factors for prognosis. These findings suggest that a subset of children with MPE may experience severe toxic or immune responses following *Mycoplasma pneumoniae* infection, damaging specific brain regions, such as the hippocampus and cerebral cortex, leading to a reduced level of consciousness or respiratory failure and significantly affecting prognosis. Therefore, further research exploring the underlying pathophysiological pathways is necessary to clarify the exact mechanisms underlying severe damage to brain tissue cells.

It is currently believed that the onset time of MPE is closely associated with the pathogenesis of *Mycoplasma pneumoniae*. Some scholars have proposed that MPE should be classified into two subtypes: early-onset (neurological symptoms occurring within 7 days after fever) and late-onset (neurological symptoms occurring more than 7 days after fever). The early-onset subtype is caused mostly by the direct invasion of brain tissue by *Mycoplasma pneumoniae*, whereas the late-onset subtype is considered to involve immune-mediated mechanisms [10, 11]. In this study, we compared early-onset and late-onset cases, and the results revealed multiple statistically significant differences in clinical characteristics and laboratory indicators between the two groups. Specifically, compared with early-onset patients, late-onset patients had a younger median age, suggesting that younger children may be more prone to delayed neurological involvement; a higher incidence of cough symptoms and severe pneumonia, indicating more severe respiratory tract lesions in late-onset patients; a significantly greater proportion of patients with elevated serum IgM levels, which may be associated with the delayed activation of immune responses induced by *Mycoplasma pneumoniae* infection; and elevated D-dimer levels, which may reflect a more severe systemic inflammatory response in late-onset patients.

The immune-mediated mechanism has been recognized as the main pathogenesis of *Mycoplasma pneumoniae*-related extrapulmonary diseases [17]. It is hypothesized that the recognition of *Mycoplasma pneumoniae* by innate immune cells and subsequent cellular activation may be the primary factors that induce severe *Mycoplasma pneumoniae* complications [18]. *Mycoplasma pneumoniae* infection can lead to disorders of innate and adaptive immunity in the host. Studies have shown that the levels of IgG, IgM, and IgA in *Mycoplasma pneumoniae*-infected patients did not increase during the 1-year observation period, suggesting that *Mycoplasma pneumoniae* infection can cause immune damage [19]. However, Patrick et al. reported that *Mycoplasma pneumoniae* can activate B lymphocytes to produce nonspecific polyclonal antibodies, which are not directly targeted against *Mycoplasma pneumoniae* [20]. In this study, serum IgG elevation was detected in 26 out of 71 children, and serum IgM elevation was detected in 20 children. Multivariate analysis revealed that elevated serum IgG was an independent risk factor associated with the prognosis of children with MPE. This finding suggests that some children infected with *Mycoplasma pneumoniae* may experience excessive activation of B lymphocytes, leading to the production of nonspecific polyclonal antibodies, which may in turn contribute to immune-mediated central nervous system damage. These observations only reflect the correlation between serum IgG levels and disease prognosis, and the specific underlying mechanisms require further investigation.

The results of the study by Fan et al. indicated that elevated CSF protein level is an independent risk factor for poor prognosis in children with MPE [1]. In our study, 43.7% of the patients had elevated CSF protein levels, but this finding was not statistically significant. This may be associated with the small number of patients with poor outcomes. The diagnosis of MPE remains problematic because of the low sensitivity of traditional microbial detection methods. In this study, *Mycoplasma pneumoniae* nucleic acid was positive in only 5.6% of children’s cerebrospinal fluid samples, and *Mycoplasma pneumoniae*-IgM was positive in 9.9% of children, which is consistent with the findings of previous studies [1, 12]. These findings suggest that *Mycoplasma pneumoniae* is rarely detected in cerebrospinal fluid, possibly because of its harsh growth conditions and limited duration of persistence in the blood. For all the children with suspected MPE in our center, viral nucleic acid testing (including respiratory viruses, enteroviruses, herpes simplex virus, etc.), CSF PCR testing, and metagenomic next-generation sequencing (mNGS) of CSF pathogens were performed to rule out viral encephalitis. Additionally, CSF antibodies related to autoimmune encephalitis (such as NMDAR and AQP4 antibodies) were detected to assist in the exclusion of autoimmune encephalitis. Through the triple diagnostic logic of “clinical symptoms + serological evidence + exclusion of other aetiologies”, the risk of overdiagnosis has been minimized. Moreover, despite the adoption of multidimensional screening methods, the lack of direct microbiological evidence in CSF may still lead to potential biases. In the future, multicenter, prospective studies combined with more sensitive detection technologies, such as mNGS, can be conducted to further optimize the diagnostic accuracy.

Abnormal EEG changes can intuitively reflect the degree of damage to brain tissue. EEG can serve as an effective tool for assessing the severity of the condition, especially in the early stage of the disease. Previous studies have reported that abnormal EEG is an independent risk factor affecting the prognosis of children with MPE [1]. In this study, only 32.4% of patients presented with abnormal EEG. This relatively low proportion may be related to the lack of EEG data in some cases, which may in turn have a certain impact on the statistical power of the prognostic factor analysis. Nevertheless, the results of univariate analysis still suggest a strong correlation between abnormal EEG and poor outcomes in children with MPE, which is consistent with the conclusions of previous relevant studies. This further supports that abnormal EEG can be used as an important reference indicator for evaluating the condition and prognosis of children with MPE.

For the treatment of MPE in children, antimicrobial agents against *Mycoplasma pneumoniae*, such as macrolides, tetracyclines, and quinolones, are typically selected in combination with appropriate immunomodulatory therapies, such as corticosteroids, IVIG, or plasma exchange, on the basis of the condition of patients [21, 22]. Currently, controversy remains regarding the efficacy and dosage of glucocorticoids. Studies have shown that once MPE is diagnosed, aggressive immunotherapy (such as glucocorticoids and IVIG) combined with antimicrobial therapy may help improve the prognosis of patients [23, 24]. In this study, 97.2% of the children received azithromycin treatment, 85.9% received glucocorticoid treatment, and 73.2% received IVIG treatment. After aggressive treatment, most children (87.3%) achieved a good prognosis. However, the analysis of prognostic factors showed no significant association between the time interval from onset to the initiation of azithromycin and glucocorticoid treatment and prognosis, indicating that no clear correlation between treatment timing and the prognosis of children with MPE was identified in this study. Notably, the number of patients with poor outcomes was small in this study, which may have limited the statistical power of the analysis. This result is merely an exploratory finding rather than a definitive conclusion. This may be related to individual differences in host immune responses or to statistical bias caused by factors such as a limited sample size and incomplete exclusion of potential confounding factors. At present, our center is conducting research on the mechanism of MPE on the basis of single-cell transcriptome sequencing, with the goal of further exploring the pathogenesis of childhood MPE and achieving more precise treatment.

In conclusion, this study revealed that most children with MPE have a good prognosis, but some still develop severe neurological sequelae. A reduced level of consciousness at disease onset, elevated serum IgG levels, and receiving invasive mechanical ventilation may serve as potential markers for poor prognosis in children with MPE. Clinically, these indicators can be used to conduct early risk stratification of children, and intensive treatment should be promptly initiated for high-risk patients, and disease progression should be closely monitored, thereby facilitating the early identification of severe cases and reducing the risk of adverse outcomes. Future research should focus on the pathogenesis of MPE, explore novel therapeutic strategies to improve the prognosis of children with MPE, and provide more robust evidence for precise clinical management.

This study has several limitations. First, this was a single-center retrospective study. Some of the enrolled children were severe cases referred from primary hospitals, which may have led to selection bias. Furthermore, the incidence of adverse outcomes in this study was relatively low, and there may be potential residual confounding factors. Third, the follow-up period was relatively short. In the future, multicenter and prospective studies, including larger cohorts and extended follow-up times, need to be carried out to verify these findings and improve the prognostic model.

## Supplementary Information

Below is the link to the electronic supplementary material.

Supplementary Material 1

## Data Availability

No datasets were generated or analysed during the current study.
