# Supplementary material for: Clinical features and short-term outcomes in children with Mycoplasma pneumoniae encephalitis
Source: BMC Infect Dis. 2026 Apr 4;26:970. doi: 10.1186/s12879-026-13253-2 (PMC13188777; doi:10.1186/s12879-026-13253-2)
Supplement: Supplementary file 1 — Supplementary Material 1 [file 12879_2026_13253_MOESM1_ESM.docx]

**Supplementary Material Table 1** Univariate analysis of factors associated with prognosis in all MPE patients (*n*= 71)

| **Parameters** | **All patients (*n*= 71)** | **Good outcome (*n*= 62)** | **Poor outcome (*n*= 9)** | ***χ*^2^** | ***P* value** |
| --- | --- | --- | --- | --- | --- |
| Age < 7 (years) | 32 (45.1) | 26 (41.9) | 6 (66.7) | 1.942 | 0.282 |
| Male | 43 (60.6) | 35 (56.5) | 8 (88.9) | 2.237 | 0.135 |
| Seizure | 25 (35.2) | 20 (32.3) | 5 (55.6) | 0.988 | 0.320 |
| Reduced level of consciousness at onset | 9 (12.7) | 4 (6.5) | 5 (55.6) | 12.971 | ＜0.001 |
| Early-onset group | 31 (43.7) | 29 (93.5) | 2 (22.2) | 1.057 | 0.304 |
| Initial WBC count ≥10 (×10^9^/L) | 38 (53.5) | 33 (53.2) | 5 (55.6) | 0.017 | 1.000 |
| CRP elevation | 34 (47.9) | 28 (45.2) | 6 (66.7) | 1.456 | 0.295 |
| LDH elevation | 25 (35.2) | 22 (35.5) | 3 (33.3) | 0.000 | 1.000 |
| Abnormal liver function | 5 (50.7) | 29 (46.8) | 6 (66.7) | 1.244 | 0.307 |
| Abnormal renal function | 18 (25.4) | 14 (22.6) | 4 (44.4) | 0.998 | 0.318 |
| D-dimer elevation | 40 (56.3) | 34 (54.8) | 6 (66.7) | 0.095 | 0.757 |
| Serum IgA elevation | 11 (15.5) | 11 (19.0) | 0 (0) | 0.711 | 0.399 |
| Serum IgG elevation | 26 (36.6) | 20 (35.1) | 6 (75.0) | 4.656 | 0.040 |
| Serum IgM elevation | 20 (28.2) | 15 (26.3) | 5 (62.5) | 2.781 | 0.166 |
| Serum IgE elevation | 35 (49.3) | 32 (57.1) | 3 (37.5) | 1.090 | 0.451 |
| Initial WBC count in CSF >5 (×10^9^/L) | 54 (76.1) | 48 (78.7) | 6 (66.7) | 0.142 | 0.706 |
| Initial protein in CSF ≥450 (mg/L) | 31 (43.7) | 30 (50.0) | 1 (12.5) | 4.002 | 0.063 |
| Time from the onset of the illness to the initiation of azithromycin treatment ≥6 (days) | 37 (52.1) | 32 (52.5) | 5 (55.6) | 0.030 | 1.000 |
| Time from the onset of the illness to the initiation of glucocorticoid treatment ≥10 (days) | 37 (52.1) | 29 (55.8) | 8 (88.9) | 2.275 | 0.131 |
| IVIG treatment | 52 (73.2) | 43 (69.4) | 9 (100.0) | 2.365 | 0.124 |
| Abnormal signal on cranial MRI | 51(71.8) | 42(67.7) | 9(100.0) | 2.605 | 0.107 |
| Basal ganglia involvement on cranial MRI | 34 (47.9) | 29 (46.8) | 5 (55.6) | 0.243 | 0.729 |
| Multiple site involvement on cranial MRI | 35 (49.3) | 30 (48.4) | 5 (55.6) | 0.162 | 0.735 |
| Abnormal EEG | 23 (32.4) | 18 (51.4) | 5 (100.0) | 4.224 | 0.061 |
| Invasive mechanical ventilation | 10 (14.1) | 5 (8.1) | 5 (55.6) | 10.987 | 0.001 |

***CRP* C-reactive protein, *CSF* cerebrospinal fluid, *EEG* electroencephalogram, *Ig* immunoglobulin, *IVIG* intravenous immunoglobulin, *LDH* lactate dehydrogenase, *MPE* *Mycoplasma pneumoniae* encephalitis, *MRI* magnetic resonance imaging, *WBC* white blood cell**
